# A comprehensive empirical comparison of hubness reduction in high-dimensional spaces

**DOI:** 10.1007/s10115-018-1205-y

**Published:** 2018-05-18

**Authors:** Roman Feldbauer, Arthur Flexer

**Affiliations:** Austrian Research Institute for Artificial Intelligence, Freyung 6/6/7, 1010 Vienna, Austria

**Keywords:** Hubness, Curse of dimensionality, Secondary distances, Classification, Nearest neighbors

## Abstract

Hubness is an aspect of the *curse of dimensionality* related to the *distance concentration* effect. *Hubs* occur in high-dimensional data spaces as objects that are particularly often among the nearest neighbors of other objects. Conversely, other data objects become *antihubs*, which are rarely or never nearest neighbors to other objects. Many machine learning algorithms rely on nearest neighbor search and some form of measuring distances, which are both impaired by high hubness. Degraded performance due to hubness has been reported for various tasks such as classification, clustering, regression, visualization, recommendation, retrieval and outlier detection. Several hubness reduction methods based on different paradigms have previously been developed. Local and global scaling as well as shared neighbors approaches aim at repairing asymmetric neighborhood relations. Global and localized centering try to eliminate spatial centrality, while the related global and local dissimilarity measures are based on density gradient flattening. Additional methods and alternative dissimilarity measures that were argued to mitigate detrimental effects of distance concentration also influence the related hubness phenomenon. In this paper, we present a large-scale empirical evaluation of all available unsupervised hubness reduction methods and dissimilarity measures. We investigate several aspects of hubness reduction as well as its influence on data semantics which we measure via nearest neighbor classification. Scaling and density gradient flattening methods improve evaluation measures such as hubness and classification accuracy consistently for data sets from a wide range of domains, while centering approaches achieve the same only under specific settings.

## Introduction

Learning in high-dimensional spaces is often challenging due to various phenomena that are commonly referred to as curse of dimensionality [4]. One well-known aspect of the curse is concentration of distances (or measure). With dimensionality approaching infinity, all distances between pairs of objects become indistinguishable [23], undermining the very concept of neighborhood-based approaches.

Hubness is a related phenomenon of the dimensionality curse: in high-dimensional spaces, some objects are closer to the global centroid (unimodal data) or local centroid (multimodal data) [44]. These objects often emerge as *hubs* with high *k*-occurrence, that is, they are among the *k*-nearest neighbors of many objects. Simultaneously, other objects are extruded from nearest neighbor lists, which makes some of these objects appear in no or few of these lists (antihubs). Due to these effects, nearest neighbor relations between any two objects become asymmetric more often in high dimensions than in low dimensions. That is, there are more unidirectional relations in high dimensions (object $\varvec{x}$ is among the nearest neighbors of $\varvec{y}$, but not vice versa). Nearest neighbor relations in high hubness regimes are prone to semantic incorrectness: Hubs propagate their encoded information too widely in corresponding distance spaces, while information carried by antihubs is essentially lost [57]. Consequently, these distance spaces do not reflect class information well, that is, semantic meaning of the data. Since intraclass distances should generally be smaller than interclass distances, nearest neighbor classification accuracy can be used as a proxy to measure semantic correctness [21].

Hubness has been identified as a detrimental factor in similarity-based machine learning, impairing several classification [44], clustering [47, 60], regression [7], graph analysis [22], visualization [17], and outlier detection [18, 19, 45] methods. Reports on affected tasks include multimedia retrieval [51], recommendation [48], collaborative filtering [25, 34], speaker verification [50], speech recognition [62], and image data classification [58].

Various strategies to mitigate detrimental effects of hubness have previously been investigated. In the course of these studies, several techniques for hubness reduction have been developed. An overview is given in Sect. 2.1. With the high number of hubness reduction methods now at hand, a comprehensive overview of their methodology and an empirical comparison of their performance is still lacking. This study aims at providing this overview and comparison. We present a comprehensive empirical evaluation of unsupervised hubness reduction methods, which showed promising results in previous studies, examining their ability to reduce hubness and whether they respect semantics of the data spaces at the same time.

This article is structured as follows: Sect. 2 reviews properties of hubness and how they may be used for hubness reduction. It also provides an overview of previous empirical comparisons. Section 3 describes the hubness reduction methods. Section 4 details the evaluation framework, data sets, evaluation measures and statistical analyses. The results of this evaluation are presented in Sect. 5 and discussed in Sect. 6. Section 7 concludes the paper and gives an outlook for future research.

This article is a substantially expanded version of a conference contribution [16]. The main extensions are the evaluation of more methods (twelve instead of four), on more data sets (50 instead of 28), an enhanced hyperparameter tuning scheme, and a more comprehensive analysis of results, including rigorous statistics.

## Related work

Hubness was first noted as a problem in automatic music recommendation [3], more specifically that certain songs were being recommended conspicuously often in nearest neighbor-based playlists. The phenomenon of hubness has then been characterized extensively in both theoretical and empirical aspects by Radovanović et al. [44]. Their publication already provided some starting points for the development of methods mitigating negative effects of hubness. Subsequently, such hubness reduction methods were designed by a number of other authors [2, 21, 26, 27, 49, 56, 59]. We summarize these concepts and methods into four categories, as outlined in the paragraphs of Sect. 2.1.

### Hubness and its reduction

Hubness is known to arise in high-dimensional data spaces. This was shown to be caused by inherent properties of data distributions, not by other effects such as finite sample sizes [44]. Data sets are often embedded in spaces of higher dimensionality than is needed to capture all their information. The minimum number of features necessary to encode this information is called intrinsic dimension (ID). More formally, ID refers to a lower-dimensional submanifold of the embedding space containing all data objects without information loss [8]. Several methods for intrinsic dimension estimation have been proposed (see, for example, Ref. [8] for a recent review). Empirical results suggest that hubness depends on a data set’s intrinsic dimension rather than the embedding dimension [44]. A later study challenges this view and argues that hubness arises due to density gradients in data sets [39], that is, spatial variations in the density of empirical data distributions. Density gradients may originate from data generating processes. Data sets consisting of points sampled from a continuous probability density function $f(\cdot )$ exhibit density gradients. Consider, for example, the bell curve-shaped PDF of a normal distribution. Additionally, density gradients emerge necessarily, when sampling regions are bounded (that is, $\exists x : f(x) = 0$), which even holds for uniform distributions otherwise not showing density gradients. Consequently, data sets sampled from uniform distributions with bounds still show density gradients [39]. With increasing dimensionality, the ratio of the size of a boundary and its encapsulated volume increases exponentially. From the viewpoint of density gradient, this explains the emergence of hubs in high-dimensional bounded data [39]. Common **dimensionality reduction** (DR) methods have been investigated in regard to hubness. Empirical results indicate principal component analysis (PCA), independent component analysis (ICA), and stochastic neighborhood embedding (SNE) to not significantly change hubness, unless the number of features falls below the intrinsic dimension of a data set [44]. In the latter case, information loss regarding pairwise distances and nearest neighbor relations may occur. Since this is an undesired effect for any neighborhood-based analysis, these DR methods are not suitable for hubness reduction. On the other hand, DR methods changing underlying pairwise distances, such as isomap or diffusion maps, do reduce hubness when retaining a number of features greater than the intrinsic dimension [44]. This finding directly motivates the adaption and development of secondary distance measures specialized on hubness reduction, as discussed under the next categories.

Objects in proximity of the sample mean of some data distribution are prone to become hubs in high-dimensional spaces, which is known as the spatial centrality of hubs [44]. For unimodal data, hubs are often close to the global data centroid. Real-world data sets are often better described as a mixture of distributions, for which hubs tend to be close to the mean of individual distributions [44]. Since the exact mixture of distributions in real-world data is often unknown, *k*-means clusters [44] and local neighborhoods [27] have previously been used to describe spatial centrality in multimodal data. On the other hand, antihubs are typically far from centers and can be considered distance-based outliers [44]. Spatial centrality can thus be described by correlation between *k*-occurrence and distance to the centroid. Reducing **spatial centrality** is another approach to reduce hubness. *Centering* (subtraction of the centroid) was proposed to eliminate hubness from text data using inner product similarities [56]. Based on this, *localized centering* was developed for hubness that may arise due to large data set size rather than high dimensionality [27]. The same authors present *DisSim*$^\mathrm{Global}$ and *DisSim*$^\mathrm{Local}$ as variants of the above, applicable to Euclidean distance spaces, and argue that hubness reduction is achieved by flattening the data density gradient [26].

Nearest neighbor relations between two objects $\varvec{x}$ and $\varvec{y}$ are considered symmetric, if $\varvec{x}$ is among the nearest neighbors of $\varvec{y}$ and vice versa. Hubness directly affects rates of symmetry with more asymmetric relations arising under high hubness conditions [49], because hubs are by definition nearest neighbors to very many data points but only one data point can be the nearest neighbor to a hub. In addition, asymmetric nearest neighbor relations offend against the pairwise stability of clusters [49], leading to wrong information propagation. For this reason, the third category of hubness reduction methods aims at **repairing asymmetric relations**. Several methods have been proposed that symmetrize these relations by transformation to secondary distance spaces, that is, they are computed from other primary distance spaces (e.g., Euclidean distances). Among these methods are *shared nearest neighbors* [32], *local scaling* [64], the *(non-iterative) contextual dissimilarity measure* [33], *mutual proximity* [49], and *simhub* [59]. Only the latter two methods were developed explicitly for hubness reduction.

Finally, the related concentration effect may be mitigated by using alternative distance measures, for example, fractional norms [23]. Analogously, **alternative distance measures** might be less prone to hubness than commonly used measures like Euclidean or cosine distances. Consequently, $\ell ^p$ norms were investigated in regard to their influence on hubness [21]. The data-dependent $m_p$-dissimilarity measure was recently presented as an alternative to geometric distances [2].

### Previous comparisons of hubness reduction

Several empirical comparisons of hubness reduction methods have been conducted previously, typically in the context of presenting new methods.

Five dimensionality reduction methods were tested for their capability to reduce hubness on three real-world data sets [44]. PCA, ICA, and SNE fail to reduce hubness, unless dimensionality is reduced below ID. Isomap and diffusion maps show some hubness reduction capability. No tests to examine whether data semantics are respected by dimensionality reduction were performed.

The local and global scaling methods mutual proximity (MP) and non-iterative contextual dissimilarity measure (NICDM) were investigated with respect to both hubness reduction and improved data semantics on thirty real-world data sets from various domains [49]. Both scaling methods showed improved performance measures on high-dimensional data sets, and no degradation on low-dimensional data sets. This is true for both hubness reduction and nearest neighbor classification. Approximate MP variants were found to perform nearly as well as full MP.

Shared nearest neighbors (SNN) was compared to local scaling and mutual proximity on six real-world data sets [20]. SNN was able to reduce hubness, though not as strongly as the other methods. Classification accuracy was improved by SNN only in three data sets, and the method thus deemed inferior to LS and MP, both of which improved accuracy in all six cases.

Simhub is a hybrid method composed of supervised (simhub$^\mathrm{PUR}$) and unsupervised (simhub$^\mathrm{IN}$) parts. Its evaluation was primarily performed for full simhub [59]. The individual component simhub$^\mathrm{IN}$ was compared to SNN on one image data set, for which it surpassed its competitor in terms of two classification measures.

Centering was compared to MP on three data sets from the text domain [56]. Both hubness and classification measures were improved for both methods to a similar extent and on par with a state-of-the-art technique. A follow-up study [27] compared localized centering (LCENT), centering, mutual proximity, local scaling, and a commute-time kernel (CTL) on four text data sets. The strongest hubness reduction was achieved by MP. LCENT and LS performed slightly better than MP in terms of classification accuracy. CTL appears to be non-effective in hubness reduction.

DisSim$^\mathrm{Local}$ was shown to outperform MP on four real-world data sets [26]. Both methods improve hubness and accuracy measures compared to DisSim$^\mathrm{Global}$ and to the Euclidean baseline.

Finding an optimal $\ell ^p$ norm was shown to improve classification on seven data sets [21]. On four data sets, LS and MP were able to further increase accuracy. SNN yielded non-competitive results over all seven data sets in that comparison.

The $m_p$-dissimilarity reduces hubness in synthetic data [2]. It was reported to do so as well in real-world data sets, but results were not provided.

A comprehensive empirical comparison overcoming several shortcomings of the above-mentioned ones is still lacking. Such a comparison must (i) evaluate all available hubness reduction methods (ii) on a large number of data sets (iii) from various domains and (iv) present appropriate statistical treatment of the results. We strive to address all of these issues in this study. Please note that we did not include commute-time kernels and dimensionality reduction in our study, since these methods performed very poorly in previous studies. We also restricted our comparison with unsupervised methods, which do not use class label information for hubness reduction. Supervised methods like simhub [59] should rather be compared to related supervised approaches like, e.g., metric learning [35], which is beyond the scope of this paper.

## Hubness reduction methods

This section reviews all unsupervised hubness reduction methods used in this paper. Some methods operate on vector data directly, others on distances between pairs of objects. Usually, Euclidean or cosine distances are used as input for the latter methods. Some methods also operate on non-metric dissimilarities or similarities. We use Euclidean distances as primary distances, unless ${{\mathrm{kNN}}}$-classification with cosine distances yields significantly better results (McNemar test, not shown for brevity). Let $B \subseteq \mathbb {R}^m$ be a non-empty data set with *n* data objects in *m*-dimensional space, that is, $b_i = ( b_{i,1}, \dots , b_{i,m} ) \in B$ for $i \in \{ 1,\dots ,n \}$. Let $\varvec{x}$, $\varvec{y}$, and $\varvec{z}$ be short-hands for three *m*-dimensional numeric vectors $b_x, b_y$, and $b_z$, respectively. Let $d : B \times B \rightarrow \mathbb {R}$ be a measure of dissimilarity. The dissimilarity between two objects $\varvec{x}$ and $\varvec{y}$ is then denoted as $d_{x,y}$. Most hubness reduction methods have tunable hyperparameters. We try to follow the notation of the original publications, and thus reuse some symbols in multiple methods. We do so only, if their meaning is closely related. For example, *k* always refers to neighborhood size, though individual methods may use nearest neighbor information differently. Descriptions of all parameters follow in the next sections.

### Measuring hubness

Before we introduce hubness reduction methods, we briefly introduce measures commonly used for describing the degree of hubness in a data set.

#### k-occurrence

The *k*-occurrence $O^k(x)$ of an object $\varvec{x}$ is defined as the number of times $\varvec{x}$ resides among the *k* nearest neighbors of all other objects in the data set. In the notion of network analysis, $O^k(x)$ is the indegree of $\varvec{x}$ in a directed ${{\mathrm{kNN}}}$ graph. It is also known as reverse neighbor count.

#### Hubness

**Fig. 1:**
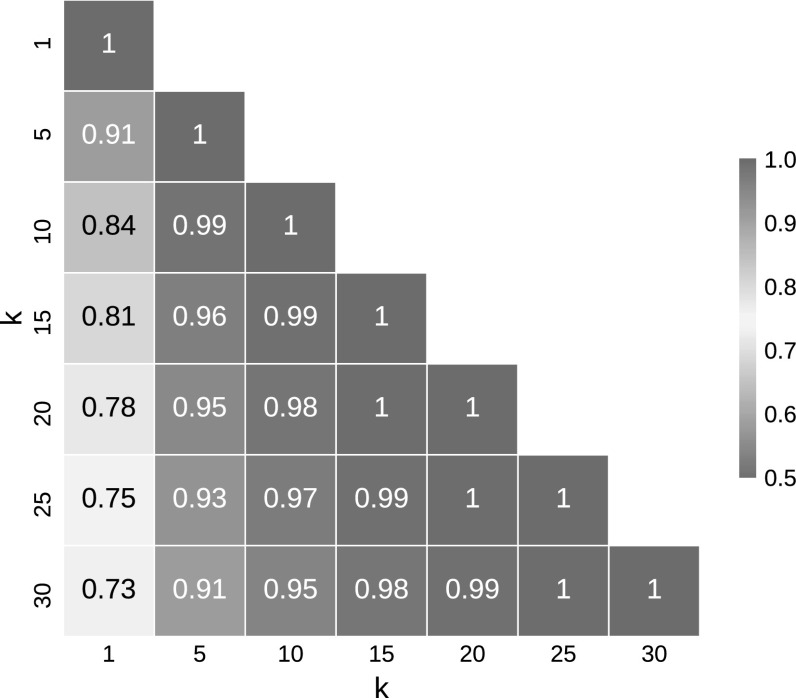
Spearman correlation of *k*-occurrence using all data sets from Table 3. For each of the 50 data sets *k*-occurrences with $k = 1, 5, 10, 15, 20, 25, 30$ are computed and correlations between *k*-occurrences with different *k* are shown

Hubness is typically measured as the skewness of the *k*-occurrence distribution [44]:

1 $$\begin{aligned} S^k = \frac{\mathbb {E}[(O^k - \mu _{O^k})^3]}{\sigma _{O^k}^3}, \end{aligned}$$

where $\mu _{O^k}$ and $\sigma _{O^k}$ denote the mean and standard deviation of the *k*-occurrence distribution, respectively. Typical values of *k* used in the literature include 1, 5, 10, and 20. Previous research indicates the choice of *k* to be non-critical. For the real-world data sets used in this paper, we observe very high correlation of *k*-occurrence among various *k* values (Fig. 1), except for $k=1$, which is less correlated. We therefore deem any values of $5 \le k \ll n$ suitable for analysis of hubness reduction and use $k=10$ for all hubness measurements in this paper.

### Methods based on repairing asymmetric relations

The following methods aim at repairing asymmetric neighbor relations. All these methods compute *secondary distances* by transforming the original primary distance (for example, Euclidean or cosine) in a data set.

#### Local scaling and the non-iterative contextual dissimilarity measure

Local scaling (LS) was proposed to improve spectral clustering performance on data of multiple scales [64]. Pairwise secondary distances are calculated as:

2 $$\begin{aligned} {{\mathrm{LS}}}(d_{x,y}) = 1 - \exp \left( -\frac{d_{x,y}^2}{\sigma _x \sigma _y} \right) . \end{aligned}$$

The scaling parameter $\sigma _x (\sigma _y)$ is set to the distance between object $\varvec{x} (\varvec{y})$ to its *k*-th nearest neighbor. LS induces increased symmetry in nearest neighbor relations by incorporating local distance information and was proposed for hubness reduction for that reason [49].

The non-iterative contextual dissimilarity measure (NICDM, [33]) is closely related to local scaling: The scaling factor of an object $\varvec{x}$ is set to the mean distance to its *k* nearest neighbors (compared to using only the *k*-th neighbor in LS). We use NICDM transformations adapted for hubness reduction [49]:

3 $$\begin{aligned} {{\mathrm{NICDM}}}(d_{x,y}) = \frac{d_{x,y}}{\sqrt{\mu _x\,\mu _y}}, \end{aligned}$$

where $\mu _x$ denotes the mean distance from object $\varvec{x}$ to its *k*-nearest neighbors (analogous for $\mu _y$ and object $\varvec{y}$). Parameter *k* in both LS and NICDM should reflect the embedding space around each object and can be tuned in order to minimize hubness.

#### Global scaling: mutual proximity

While LS and NICDM use local distance statistics to enforce symmetric neighborhoods, mutual proximity (MP, [49]) incorporates information of all pairwise distances in the data set to achieve the same. Let *X* be a random variable of distances between $\varvec{x}$ and all other objects in the data set (analogously for *Y* and $\varvec{y}$), and *P* the joint probability density function, then

4 $$\begin{aligned} {{\mathrm{MP}}}(d_{x,y}) = P ( X> d_{x,y} \cap Y > d_{y,x} ). \end{aligned}$$

Secondary distances are calculated as the complement of the joint probability of two objects being nearest neighbors to each other (i.e., $1 - {{\mathrm{MP}}}$). To allow for this probabilistic view, MP models the distances $d_{x,i \in \{1,\dots ,n\} \setminus x}$ between an object $\varvec{x}$ and all other objects with some distribution. When using the empirical distance distribution, mutual proximity between two objects $\varvec{x}$ and $\varvec{y}$ is calculated by counting objects whose distances to both $\varvec{x}$ and $\varvec{y}$ are greater than *d*(*x*, *y*):

5 $$\begin{aligned} {{\mathrm{MP}}}(d_{x,y}) = \frac{|\{ j : d_{x,j}> d_{x,y}\} \cap \{ j : d_{y,j} > d_{y,x}\}|}{n - 2}. \end{aligned}$$

Compared to the formula in the Ref. [49], we added a subtrahend to the denominator to account for identity distances. This influences the normalization to the [0, 1] range but does not change neighborhood order, hubness, or nearest neighbor classification.

In the framework of MP, distances can also be modeled with any (continuous) distribution. This is especially useful, when the user has prior knowledge of the given data domain. Additionally, if *X* and *Y* are assumed to be independent, Formula 4 simplifies to

6 $$\begin{aligned} {{\mathrm{MP}}}^\text {I}(d_{x,y}) = P(X> d_{x,y}) \cdot P(Y > d_{y,x}). \end{aligned}$$

These approximations simplify calculations and decrease the computational complexity of MP. The Gaussian-based mutual proximity variant (MP$^\mathrm{GaussI}$) models the distances of each object $\varvec{x}$ to all other objects with a normal distribution ($X \sim \mathcal {N}(\mu , \sigma ^2)$). Parameters $\mu _x$ and $\sigma ^2_x$ can be estimated with the sample mean $\hat{\mu }_x$ and variance $\hat{\sigma }^2_x$:

7 $$\begin{aligned} \mathcal {N}_{x} \backsim \qquad \hat{\mu }_{x} = \frac{1}{n-1} \sum _{i=1, i \ne x}^{n} d_{x, i}, \qquad \hat{\sigma }_{x}^2 = \frac{1}{n-1} \sum _{i=1, i \ne x}^{n} (d_{x,i} - \hat{\mu }_{x})^2 \end{aligned}$$

Compared to Ref. [49], we exclude self-distances $d_{x,x}$ from parameter estimation. This should presumably improve the approximation, since self-distances are not informative. Secondary distances based on MP$^\mathrm{GaussI}$ are calculated as

8 $$\begin{aligned} {{\mathrm{MP}}}^\text {GaussI}(d_{x,y}) = \hbox {SF}(d_{x,y}, \hat{\mu }_x, \hat{\sigma }^2_x) \cdot \hbox {SF}(d_{y,x}, \hat{\mu }_y, \hat{\sigma }^2_y), \end{aligned}$$

where $\hbox {SF}(d, \mu , \sigma ^2) = 1 - \hbox {CDF}(d, \mu , \sigma ^2)$, that is, the survival function (complement to the cumulative density function) at value *d* given the indicated distribution.

#### Shared nearest neighbors and simhub

A shared neighborhood is the intersection of the nearest neighbor sets of two objects [32]. Secondary distances based on shared nearest neighbors (SNN) increase pairwise stability and relation symmetry, which is considered beneficial for hubness reduction [20]. SNN similarities are calculated as:

9 $$\begin{aligned} {{\mathrm{SNN}}}(x,y) = \frac{| {{\mathrm{kNN}}}(x) \cap {{\mathrm{kNN}}}(y) |}{k}, \end{aligned}$$

where ${{\mathrm{kNN}}}(\cdot )$ is the set of the *k*-nearest neighbors of some object.

Simhub [59] is a shared neighbors approach that weights shared neighbors $\varvec{z}$ by informativeness (increasing weights of rare neighbors) and purity (penalizes neighborhoods with inconsistent class labels). Both weights may be used simultaneously (simhub) or separately (simhub$^\mathrm{IN}$ and simhub$^\mathrm{PUR}$ for informativeness and purity, respectively). Simhub is a supervised method when using purity weights. We thus restrict our evaluation to the unsupervised simhub$^\mathrm{IN}$:

10 $$\begin{aligned} \text {simhub}^\text {IN}(x,y)= & {} \frac{\sum _{z \in ({{\mathrm{kNN}}}(x) \cap {{\mathrm{kNN}}}(y))}I_n(z)}{k \cdot \max I_n}, \nonumber \\ I_n(z)= & {} \log \frac{n}{O^k(z)+1}, \quad \max I_n = \log n \end{aligned}$$

where $I_n(z)$ is the occurrence informativeness of a shared neighbor $\varvec{z}$ in a data set of size *n*. The neighborhood radius *k* can be tuned in both SNN-based methods to minimize hubness. Computing $1 - {{\mathrm{SNN}}}$, or $1 - \text {simhub}$ turns the similarities into distances.

### Methods based on spatial centrality reduction and density gradient flattening

Centering approaches aim at reducing spatial centrality, and use modified inner product similarities to span distance spaces. Global and local DisSim try to flatten density gradients, and construct dissimilarities from squared Euclidean distances.

#### Centering and localized centering

Centering is a widely used preprocessing step that shifts vectors ($\varvec{x}$) so that the space origin coincides with the global centroid ($\bar{c}$). Centering dissimilarities can be calculated as

11 $$\begin{aligned} \text {CENT}(x,y) = - \langle x-\bar{c}, y-\bar{c} \rangle , \end{aligned}$$

where $\langle \cdot , \cdot \rangle$ is the inner product of two vectors. The method was proposed for hubness reduction in the context of natural language processing [56]. Centering moves the centroid to the origin. Inner product dissimilarities between any object and the origin (zero vector) are uniformly zero. Centering effectively eliminates spatial centrality in inner product spaces, which should reduce hub emergence. Following this idea, localized centering was developed [27]: Instead of shifting the whole vector space, LCENT is a dissimilarity measure based on *global affinity* (mean similarity between an object $\varvec{x}$ and all other objects) and *local affinity* (mean similarity between $\varvec{x}$ and its *k* nearest neighbors):

12 $$\begin{aligned} {{\mathrm{LCENT}}}(x,y) = - \langle x, y \rangle + \langle x, c_k(x) \rangle ^{\gamma }, \end{aligned}$$

where $c_k(x)$ denotes the local centroid among the *k* nearest neighbors of $\varvec{x}, \gamma$ is a parameter controlling the penalty introduced by the second term, and the leading negative sign indicates dissimilarities. LCENT dissimilarities are not guaranteed to be positive. Parameters $\gamma$ and *k* can be tuned to minimize hubness.

#### Global and local dissimilarity measures

The above-described centering approaches have no effect on Euclidean distances. As an alternative, two dissimilarity measures were introduced [26]: They reduce hubness by flattening the density gradient and thus eliminate spatial centrality in commonly used Euclidean spaces. The global variant DisSim$^\mathrm{Global}$ (DSG) removes *sample-wise centrality* of two objects $\varvec{x}$ and $\varvec{y}$:

13 $$\begin{aligned} {{\mathrm{DSG}}}(x,y) = \Vert x - y \Vert ^2_2 - \Vert x - c\Vert ^2_2 - \Vert y - c\Vert ^2_2, \end{aligned}$$

where *c* is the global centroid and $\Vert \cdot \Vert ^2_2$ indicates the squared Euclidean norm.

The local variant DisSim$^\mathrm{Local}$ (DSL) is free from the assumption that all instances in the data set come from the same distribution: Instead of subtracting the global centroid, local centroids are estimated as $c_k(x) = \frac{1}{k}\sum _{x' \in {{\mathrm{kNN}}}(x)} x'$, where ${{\mathrm{kNN}}}(x)$ is the set of *k*-nearest neighbors of $\varvec{x}$, and substitution in Formula 13 yields:

14 $$\begin{aligned} {{\mathrm{DSL}}}(x,y) = \Vert x - y \Vert ^2_2 - \Vert x - c_k(x)\Vert ^2_2 - \Vert y - c_k(y)\Vert ^2_2. \end{aligned}$$

Parameter *k* can be tuned to minimize hubness.

### Hubness-resistant dissimilarity measures

The methods described in this section try to avoid hubness by using alternative distance measures between data objects.

#### Choosing $\ell ^{p}$ norms and the $m_p$-dissimilarity measure

Euclidean distances correspond to a special case of the family of $\ell ^p$ norms (also known as Minkowski norms) with $p=2$. The effect of using norms with $p\ne 2$ in the context of *hubness* has been investigated previously [21]. An $\ell ^p$ norm of a vector $(\varvec{x} - \varvec{y})$ can be interpreted as a dissimilarity between $\varvec{x}$ and $\varvec{y}$ and is calculated as follows:

15 $$\begin{aligned} d^{p}\left( x,y\right) = \left( \sum _{i=1}^m | x_i - y_i |^p\right) ^{1/p} \end{aligned}$$

For $0<p<1$ the resulting Minkowski norms (also called fractional norms) do not guarantee the triangle inequality. Consequently, they do not constitute full distance metrics. The parameter *p* can be tuned to minimize hubness. In this work, we evaluate $\ell ^p$ norms with $p = 0.25, 0.5, ..., 5$ (as in Ref. [21]) and ten values randomly selected from ]0, 5[.

A data-dependent dissimilarity measure was recently derived from $\ell ^{p}$ norms [2]. The $m_p$-dissimilarity takes into account data distributions by estimating the probability mass $|R_i(x, y)|$ in a region *R* around $\varvec{x}$ and $\varvec{y}$ in each dimension *i*:

16 $$\begin{aligned} m_p(\varvec{x}, \varvec{y}) = \left( \frac{1}{m}\sum _{i=1}^m \left( \frac{|R_i(x, y)|}{n} \right) ^p\right) ^{1/p}, \quad |R_i\left( x, y\right) | = \sum _{q=l}^u |h_{i q}| \end{aligned}$$

That is, all objects are binned in each dimension. Let $h_{il}$ and $h_{iu}$ be the bins that contain $\hbox {min}(x_i, y_i)$ and $\hbox {max}(x_i, y_i)$, respectively. The probability data mass $|R_i|$ is then estimated by counting the objects in all bins from $h_{il}$ to $h_{iu}$. $R_i$ replaces the geometric distance used in $\ell ^p$ norms. Dissimilarities are thus increased in dense regions and decreased in sparse regions.

### Time and space complexity

Hubness reduction is expensive due to calculation of distances between all pairs of objects. Table 1 lists time complexity of all methods. All methods applied on data vectors require $\mathcal {O}(n^2 m)$ time^1^. Since their prefactors differ considerably, timings for two synthetic data sets of increasing size and dimensionality are also provided. Methods applied on data distances require $\mathcal {O}(n^2)$ or $\mathcal {O}(n^3)$ time in addition to $\mathcal {O}(n^2 m)$ time for preprocessing primary distances. All methods require $\mathcal {O}(n^2)$ space for returning the distance matrix. For primary distances in data sets with $m > n$ this is dominated by the memory requirement of the input vectors $\mathcal {O}(n m)$. Intermediate steps typically require $\mathcal {O}(n)$ space, except for $m_p$-dissimilarity, which requires $\mathcal {O}(b^2 m)$ for distances between all pairs of bins in each dimension, where *b* is the number of bins, and $b \ll n$.

**Table 1:** Time complexity for computing distances between all pairs of objects and timings for two synthetic data sets ($B_{1 000}$ with $n=m=1 000$ and $B_{10 000}$ with $n=m=10 000$). Timings were performed with the Hub-Toolbox for Python (see Sect. 3.6) on a single core of an Intel Core i5-6500 CPU 3.20GHz with 15.6 GiB main memory

	Input data	Time complexity	Time (s) $B_{1000}$	Time (s) $B_{10000}$	Parameters
Eucl	Vectors	$\mathcal {O}(n^2 m)$	0.1	26	–
cos	Vectors	$\mathcal {O}(n^2 m)$	0.3	48	–
MP	Distances	$\mathcal {O}(n^3)$	1.6	1382	–
MP$^\mathrm{GaussI}$	Distances	$\mathcal {O}(n^2)$	0.2	8	–
LS	Distances	$\mathcal {O}(n^2)$	$<\, 0.1$	4	$k=10$
NICDM	Distances	$\mathcal {O}(n^2)$	$<\, 0.1$	3	$k=10$
SNN	Distances	$\mathcal {O}(n^3)$	0.7	754	$k=10$
simhub$^\mathrm{IN}$	Distances	$\mathcal {O}(n^3)$	2.5	2510	$s=10$
CENT	Vectors	$\mathcal {O}(n^2 m)$	0.1	33	–
LCENT	Vectors	$\mathcal {O}(n^2 m)$	0.5	71	$\kappa =10, \gamma =1.5$
DSG	Vectors	$\mathcal {O}(n^2 m)$	0.2	46	–
DSL	Vectors	$\mathcal {O}(n^2 m)$	0.3	51	$k=10$
$\ell ^p$ norm	Vectors	$\mathcal {O}(n^2 m)$	29.8	30018	$p=1.5$
$m_p$-dissim	Vectors	$\mathcal {O}(n^2 m)$	75.8	63143	$p=1.5$,
					$$n_\text {bins}=200$$

### OFAI Hub-Toolbox

The availability of machine learning algorithms not only as formulas, but also as working code in reference implementations allows easy reproducibility and applicability of methods. Consequently, all methods described in this publication are available as part of a free open source software package for the Python programming environment. The Hub-Toolbox is easily installable from the PyPI package repository^2^ and licensed under GNU GPLv3. Please visit the GitHub page^3^ for source code, development versions, issue tracking, and contribution possibilities. A MATLAB version of the Hub-Toolbox providing core functionality is also available on GitHub.^4^

**Fig. 2:**
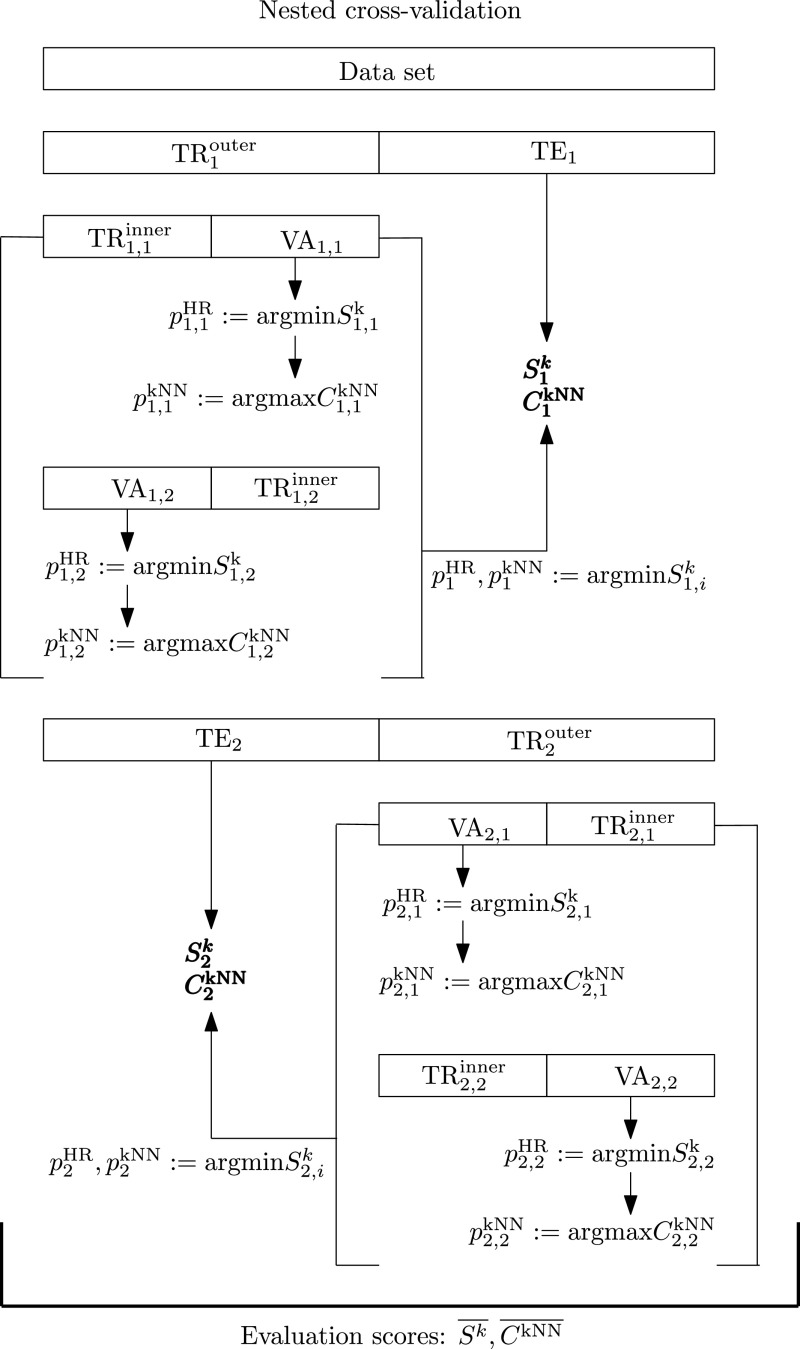
Nested cross-validation scheme. Twofold inner and twofold outer loops are depicted for better presentability. The actual evaluation is performed as tenfold/tenfold nested CV. Abbreviations: CV...cross-validation, TR...training set, VA...validation set, TE...test set, $\overline{S^k}$...mean hubness, $\overline{C^{{{\mathrm{kNN}}}}}$...mean accuracy. Text superscripts indicate outer/inner CV, subscript indices display number of outer fold and inner fold

## Evaluation

The evaluation strategy focuses on two indicators: (i) hubness is measured as the skewness of *k*-occurrence distribution (see Sect. 3.1); (ii) *k*-nearest neighbor classification accuracy $C^{{{\mathrm{kNN}}}}$ is used to measure the degree of correct data semantics in primary and secondary distance spaces. The neighborhood size parameter *k* and the weighting mode for *k*-nearest neighbor classification are selected in a nested cross-validation scheme (see Fig. 2). Weighting can be distance-based, that is, neighbors are weighted by their inverse distance during prediction, giving more influence to closer neighbors. Otherwise, nearest neighbors have uniform weights, and prediction is a majority vote among them. Ties are resolved by the nearest neighbor.

Baseline accuracy (column $C^{{{\mathrm{kNN}}}}$ in Table 3) is obtained in a cross-validation procedure as described in Sect. 4.1. Parameter *k* is selected by maximizing $C^{{{\mathrm{kNN}}}}$. This is performed using both Euclidean and cosine distances. For each data set, $C^{{{\mathrm{kNN}}}}$ is reported for the distance measure that yielded higher accuracy as indicated in column ‘d’ in Table 3.

### Evaluation scheme

In this section, we evaluate the hubness reduction methods described in Sect. 3 with regard to improved hubness and data semantics. We follow a standard procedure for comparing classifiers, preprocessing, or postprocessing steps over multiple data sets [14]. The procedure requires ’reliable’ scores, that is, they must come from an evaluation scheme with sufficiently many experiments on each data set, which should ideally be performed on the same random samples for each evaluated method.

**Table 2:** Hyperparameters and selection ranges for hubness reduction methods (first twelve rows) and ${{\mathrm{kNN}}}$ classification (last row). The methods have one or two parameters, or are parameter-free. Ranges of numerical parameters are subsets of $\mathbb {N}^+$, or $\mathbb {R}^+$ as indicated by decimal points

	Parameter 1	Range	Parameter 2	Range
MP	–	–	–	–
MP$^\mathrm{GaussI}$	–	–	–	–
LS	k	[1, 50]	–	–
NICDM	k	[1, 50]	–	–
SNN	k	[1, 50]	–	–
simhub$^\mathrm{IN}$	s	[1, n]	–	–
CENT	–	–	–	–
LCENT	$\kappa$	[5, 100]	$\gamma$	]0., 5.]
DSG	–	–	–	–
DSL	$\kappa$	[1, n]	–	–
$\ell ^p$ norm	p	]0., 5.]	–	–
$m_p$-dissim	p	]0., 5.]	n_bins_	[10, $\min (\frac{n}{2}, 200)$]
${{\mathrm{kNN}}}$	k	[1, 10], 15, 20, 25, 30, 40, 50, 100	Weights	{Uniform, distance}

We evaluate twelve hubness reduction methods on fifty data sets in a nested cross-validation (CV) scheme [9] as depicted schematically in Fig. 2 (for twofold CV for reasons of better presentability). Each data set is first split into outer training set (TR$^\mathrm{outer}$) and test set (TE) in a tenfold outer cross-validation. TR$^\mathrm{outer}$ is then split into an inner training set (TR$^\mathrm{inner}$) and a validation set (VA) in a tenfold inner cross-validation. On both levels, the data is split randomly and stratified based on the class labels. The splits are identical for all methods to meet the requirements of the comparison procedure. In the inner CV we first find optimal hyperparameter values for hubness reduction, then an optimal *k* and weighting mode for ${{\mathrm{kNN}}}$ classification. We perform randomized hyperparameter search [5], that is, we draw hyperparameter values uniformly and randomly from a predefined range, until a certain budget of samples is consumed.

Specifically, randomized hyperparameter search is performed to minimize hubness ($\min {S^k}$) on the validation set VA in each inner loop *i* of each outer loop *o* for each hubness reduction method. Table 2 lists the parameter ranges used in the optimization steps. A budget of 30 hyperparameter values is used (or 30 pairs of values in case two hyperparameters are being optimized), and the best value (pair) is denoted as $p^{\text {HR}}_{o,i}$. This step is omitted for MP, CENT, and DSG, for which there are no hyperparameters to tune. Secondary distances between objects in VA and TR$^\mathrm{inner}$ are then calculated using hubness reduction with optimal hyperparameters $p^{\text {HR}}_{o,i}$. Subsequently, classification accuracy is maximized ($\max {C^{{{\mathrm{kNN}}}}}$) on VA in the inner loop using a ${{\mathrm{kNN}}}$ classifier. The best pair of hyperparameter values (*k* and weighting mode) from a budget of ten is denoted as $p^{{{\mathrm{kNN}}}}_{o,i}$. For each outer fold *o*, the best hyperparameters $p^{\text {HR}}_{o}$ and $p^{{{\mathrm{kNN}}}}_{o}$ are selected from the inner fold showing lowest hubness among all inner folds $i=1\dots 10$.

The hubness reduction methods are scored on TE using these optimized hyperparameters, that is, $p^{\text {HR}}_{o}$ for a given hubness reduction method, and $p^{{{\mathrm{kNN}}}}_{o}$ for ${{\mathrm{kNN}}}$ classification. We calculate mean hubness $\overline{S^k}$ and mean ${{\mathrm{kNN}}}$ classification accuracy $\overline{C^{{{\mathrm{kNN}}}}}$ over ten outer folds $o=1\dots 10$. Given the evaluation strategy, these scores are assumed to be robust and unbiased performance estimates. The hubness reduction methods are then compared using the nonparametric Friedman test on both measures, independently. If significant differences are found among the methods, the post hoc Nemenyi test is used to determine the best performing method.

**Table 3:** Overview of 50 data sets from public machine learning repositories (*Source*) ordered by ascending hubness ($S^{k=10}$) of the indicated distance space (*d*)

#	Source	Name	Cls.	*n*	*m*	$m_{mle}$	*d*	$C^{{{\mathrm{kNN}}}}$	$S^{k=10}$
1	UCI	Opportunity activity recognition	5	$^\mathrm{*}$2000	238	2	$\ell ^2$	0.8995	− 0.1156
2	LibSVM	Fourclass (sc)	2	862	2	2	$\ell ^2$	1.0000	0.1528
3	LibSVM	Liver-disorders (sc)	2	345	6	4	$\ell ^2$	0.6260	0.1795
4	OpenML	Spectrometer	2	531	101	5	$\ell ^2$	0.9661	0.1903
5	UCI	Mice protein expression	8	1080	77	2	cos	0.9787	0.1961
6	LibSVM	Australian	2	690	14	2	$\ell ^2$	0.6928	0.2011
7	UCI	Chronic kidney disease	2	400	24	2	cos	0.7400	0.2745
8	UCI	Parkinson speech	2	1208	26	3	$\ell ^2$	0.6755	0.3932
9	UCI	Arcene	2	100	10000	8	$\ell ^2$	0.7500	0.4428
10	LibSVM	Breast-cancer (sc)	2	683	10	1	$\ell ^2$	0.9693	0.5686
11	LibSVM	Heart (sc)	2	270	13	3	$\ell ^2$	0.8222	0.5734
12	LibSVM	Colon-cancer	2	62	2000	10	$\ell ^2$	0.7742	0.5950
13	LibSVM	Diabetes (sc)	2	768	8	5	cos	0.7656	0.5950
14	UCI	mfeat-karhunen	10	2000	64	7	$\ell ^2$	0.9755	0.6898
15	KR	Ovarian-61902	2	253	15154	8	$\ell ^2$	0.9447	0.7603
16	OpenML	semeion	10	1593	256	12	$\ell ^2$	0.9165	0.8012
17	UCI	mfeat-factors	10	2000	216	6	$\ell ^2$	0.9570	0.8193
18	LibSVM	Duke (train)	2	38	7129	11	$\ell ^2$	0.7105	0.8275
19	CP	c224a web	14	224	1244	21	cos	0.9286	0.8345
20	LibSVM	German numbers (sc)	2	1000	24	5	$\ell ^2$	0.7320	0.8835
21	UCI	mfeat-pixels	10	2000	240	9	$\ell ^2$	0.9785	0.9642
22	KR	amlall	2	72	7129	11	$\ell ^2$	0.9167	1.1655
23	LibSVM	Sonar (sc)	2	208	60	5	$\ell ^2$	0.8462	1.1798
24	UCI	Student alcohol consumption	5	1044	56	4	$\ell ^2$	0.4157	1.2105
25	LibSVM	Splice (sc)	2	1000	60	7	cos	0.7720	1.2288
26	KR	Lungcancer	2	181	12533	10	$\ell ^2$	1.0000	1.2483
27	UCI	p53 mutants	2	1430	5408	6	cos	0.9294	1.2574
28	Corel	corel1000	10	1000	192	7	$\ell ^2$	0.6820	1.4179
29	UCI	Arrhythmia	13	452	279	10	$\ell ^2$	0.5951	1.4994
30	LibSVM	rcv1.multiclass	42	$^\mathrm{*}$2000	47236	10	cos	0.7550	1.5359
31	UCI	Reuters-transcribed	10	201	2730	23	$\ell ^2$	0.5672	1.6147
32	LibSVM	Ionosphere (sc)	2	351	34	5	$\ell ^2$	0.8917	1.6763
33	OpenML	OVA uterus	2	1545	10936	13	$\ell ^2$	0.9346	1.7759
34	OpenML	Lymphoma	11	96	4026	9	$\ell ^2$	0.8646	1.8785
35	UCI	Farm ads	2	4143	54877	1	cos	0.8938	1.9327
36	UCI	Gisette	2	6000	5000	51	cos	0.9783	1.9667
37	OpenML	AP breast ovary	2	542	10936	14	$\ell ^2$	0.9004	1.9812
38	OpenML	Hepatitis C	3	283	54621	25	$\ell ^2$	0.8975	2.1221
39	UCI	Dorothea	2	800	100000	253	cos	0.9375	2.3578
40	UCI	CNAE-9	9	1080	856	5	cos	0.8713	2.5492
41	UCI	Dexter	2	300	20000	34	$\ell ^2$	0.8667	3.3307
42	MLDATA	DMOZ	5	1329	10630	5	cos	0.4981	3.6351
43	UCI	Amazon commerce reviews	50	1500	10000	11	cos	0.3573	4.1013
44	LibSVM	Protein	3	$^\mathrm{*}$2000	357	34	cos	0.5845	4.2757
45	PaBo	Movie-reviews	2	2000	10382	44	$\ell ^2$	0.7935	4.3452
46	UCI	Mini-newsgroups	20	2000	8811	16	$\ell ^2$	0.8330	4.3705
47	LibSVM	Sector	104	$^\mathrm{*}$2000	55197	11	cos	0.7015	5.5795
48	OpenML	Wap.wc	20	1560	8460	12	cos	0.5045	9.3380
49	CP	c1ka-twitter	17	969	49820	44	cos	0.3633	10.7119
50	LibSVM	dna	3	2000	180	5	$\ell ^2$	0.8790	15.5188

The data sets are characterized by their *Name*, number of classes (*Cls.*), instances (*n*), features (*m*), and their estimated intrinsic dimension ($m_{mle}$) [36]. Column $C^{{{\mathrm{kNN}}}}$ reports baseline nearest neighbor classification performance (see Sect. 4). Asterisks indicate random samples as discussed in Sect. 4.2

### Data sets

We evaluate the previously described methods on 50 different public machine learning data sets. The selection of data sets was motivated primarily by the observed degree of hubness: Data sets with high hubness were selected in order to exploit the full potential of hubness reduction methods. Several low hubness data sets were also added to the collection. We did not expect substantial performance boosts in these cases. However, we use these data sets to investigate possible adverse effects of hubness reduction when there is hardly anything to reduce. Please note that some low-dimensional data sets were added for the very same reason. Table 3 contains details about the data sets, 28 of which have already been used in a previous study. Additional 22 data sets were obtained from four public machine learning repositories.

- Already used in a previous study [49]: *arcene*, *amlall*, *gisette*, *mfeat-factors*, *mfeat-karhunen*, *mfeat-pixels*, *heart*, *sonar*, *dexter*, *mini-newsgroups*, *dorothea*, *lungcancer*, *reuters-transcribed*, *ovarian 61902*, *australian*, *diabetes*, *german numbers*, *liver-disorders*, *breast-cancer*, *duke (train)*, *colon-cancer*, *fourclass*, *ionosphere*, *splice*, *c1ka-twitter*, *c224a-web*, *corel1000*, *movie-reviews*. Please note that *ballroom* and *ismir2004* were omitted, because they use symmetrized Kullback–Leibler divergence, which is non-trivial to combine with some of the methods evaluated here.
- UCI Machine Learning Repository [38]: *Parkinson Speech Dataset with Multiple Types of Sound Recordings* [46], *Amazon Commerce reviews* [55], *p53 Mutants* [13], *CNAE-9* [12], *Student Alcohol Consumption* [42], *Arrhythmia* [24], *Farm Ads* [41], *Mice Protein Expression* [28], *Opportunity Activity Recognition* [11], *Chronic Kidney Disease* [53]
- LibSVM [10]: *dna* [30], *protein* [63], *sector* [40], *rcv1.multiclass* [37]
- OpenML [61]: *Semeion Handwritten Digit* [52], *AP Breast Ovary* and *OVA Uterus* [54], *wap.wc*, *hepatitisC*, *Lymphoma* and *Spectrometer*
- MLdata [29]: *DMOZ* [15]

All data sets were downloaded from the sources indicated above and split into feature and label vectors according to their individual descriptions. Euclidean and cosine distances were calculated with the SciPy package for Python. Missing values were imputed with the median strategy over all instances in the following six data sets: Mice Protein Expression, Opportunity Activity Recognition, p53 Mutants, Chronic Kidney Disease, Arrhythmia, Lymphoma. For exceptionally large data sets ($n > 10000$), a stratified random sample of 2000 instances was drawn, preserving the percentage of instances for each class. This applies to the following four data sets marked with asterisks in column *n* of Table 3: Opportunity Activity Recognition, rcv1.multiclass, protein, sector. The data set p53 Mutants has highly skewed class distribution ($<1\%$ negatives). To reduce this skewness, all negative instances were kept, but positive instances were sampled at random until they made up $90\%$ of the new subset. The preexisting train-test-splits of Parkinson Multiple Sound Recordings were ignored and merged into a single data set. Categorical features of the Student Alcohol Consumption data set were transformed with a One-Hot-Encoder, i.e., to multiple binarized features. The Chronic Kidney Disease ARFF file contains several formatting errors (like, e.g., tabs after comma or at EOL, double commas) that hinder import into the evaluation framework. These errors were corrected manually. The SVMlight-styled file of the protein data set was missing leading zeros for floating point values in [0.0, 1.0). These were added manually.

## Results

**Fig. 3:**
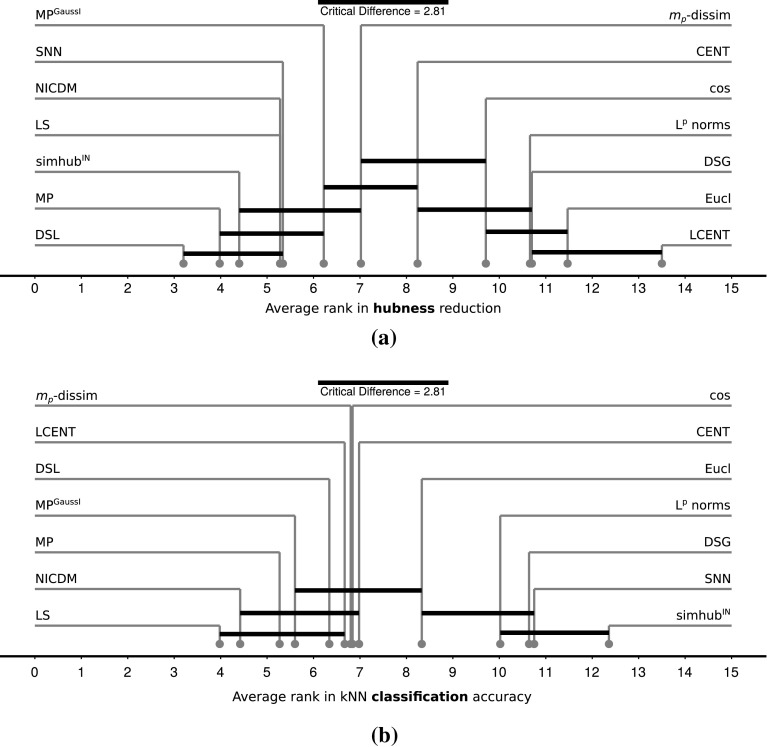
Critical difference plots of average ranks in **a** hubness reduction and **b** nearest neighbor classification accuracy $C^{{{\mathrm{kNN}}}}$ for twelve hubness reduction methods plus two baseline distances (Eucl, cos). Low ranks indicate good performance. See Sect. 5 for details

We found significant overall differences between the evaluated methods, both in terms of hubness reduction (Friedman $p < .000$) and nearest neighbor classification performance (Friedman $p < .000$). To analyze differences in more detail, we use critical difference plots ([14], see Fig. 3a, b) and post hoc Nemenyi tests. A critical difference (CD) plot shows the average ranks achieved by the competing methods across all data sets. When comparing all methods against each other, groups of methods not showing significant differences are connected with a black bar. In addition, this critical difference (CD bar length) is shown above the graph. It depends on the number of compared models (methods), the number of measurements (data sets), and the confidence level.

### Performance ranking

The CD plot for hubness reduction comparing twelve methods plus two baseline distances (Euclidean, cosine) is shown in Fig. 3a. Well-performing methods have a low average rank, that is they reduce hubness more strongly than other methods on many data sets. Any two methods with a rank difference of at least 2.81 (CD bar length) perform significantly different according to the Nemenyi test ($K=14$ models, $N=50$ measurements, $\alpha =.05$). That is, methods connected by a black bar do not differ significantly. To give one concrete example, CENT yielded significantly lower hubness than Eucl, but it does not compared to cos. Therefore, CENT is connected to cos with a CD bar, but not to Eucl. On a more general note, the post hoc Nemenyi tests show that nearly all methods reduce hubness compared to baseline distances Eucl and cos, except for using $\ell ^p$ norms, DSG, and LCENT. Method LCENT even shows increased hubness on average. Strongest hubness reduction was achieved by DSL, followed without significant rank differences by MP, simhub$^\mathrm{IN}$, LS, NICDM, and SNN.

The CD plot for nearest neighbor classification performance again comparing twelve methods plus two baseline distances (Euclidean, cosine) is shown in Fig. 3b. Regarding classification performance, the Nemenyi test reveals two coarse groups of methods: eight of them perform better or at least as well as the baselines, while four methods perform worse. Among the low rank (that is, ‘good’) methods, LS, NICDM, and MP yield significantly better results than the Euclidean baseline. Compared to both baselines, this is solely achieved by LS. The CD plot reveals, however, that the best six methods (aforementioned plus MP$^\mathrm{GaussI}$, DSL, LCENT) are ranked within the critical distance, that is, no significant difference in their performance given the evaluation setup was found. Both centering variants as well as $m_p$-dissimilarity yield results very similar to the baselines. The high rank (that is, ‘bad’) methods are significantly worse than using cosine distances, and only simhub$^\mathrm{IN}$ is also worse than using Euclidean distances.

Overall, local (LS, NICDM) and global scaling (MP) methods and DSL perform well both in regard to hubness reduction and classification performance, and appear to be the most promising hubness reduction methods. Shared neighbors methods (SNN, simhub$^\mathrm{IN}$) yield distances with reduced hubness, but also impaired classification performance, indicating that their secondary distance space does not respect the semantic meaning of the primary distance space. Localized centering (LCENT) on the other hand showed reasonable classification performance, though it increased hubness on average, instead of reducing it. DSG and $\ell ^p$ norms have hardly any influence on both evaluation measures.

**Fig. 4:**
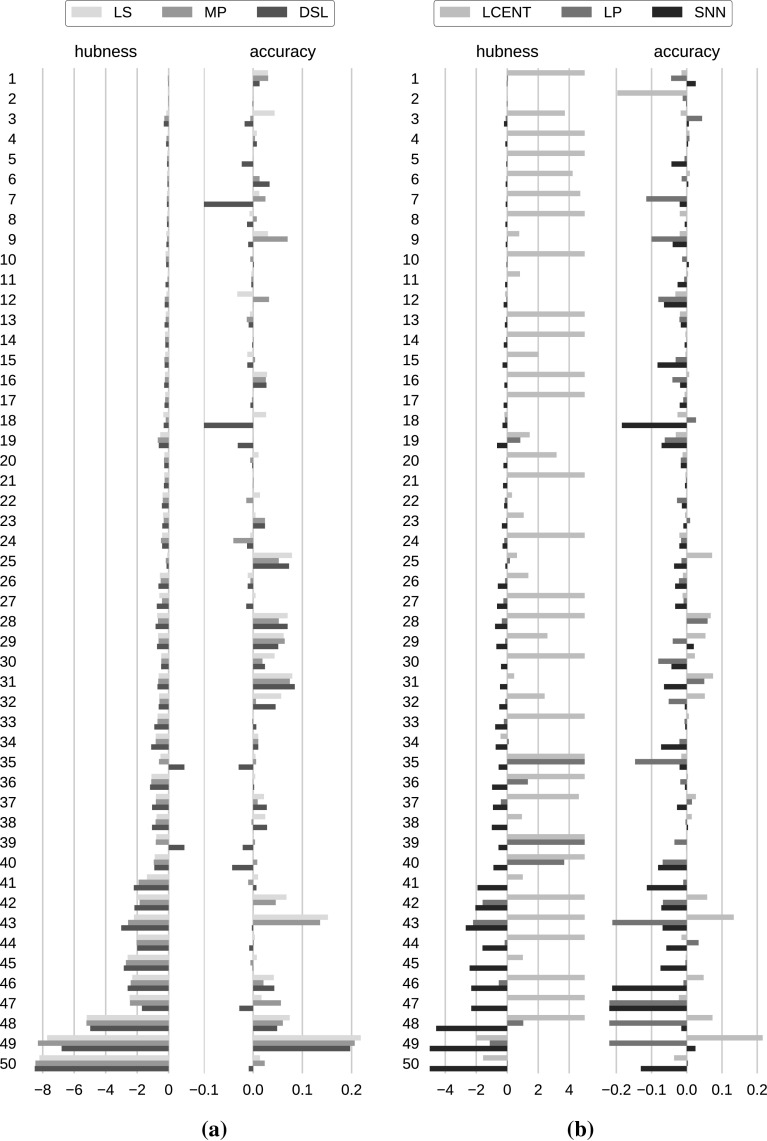
Absolute change of hubness and ${{\mathrm{kNN}}}$ classification accuracy per data set for each hubness reduction method compared to baseline (cf. Sect. 4). Improvements are indicated by negative values of hubness change, and by positive values of accuracy change. Accuracy differences can range from -1 to 1, where, e.g., 0.1 refers to a performance increase of ten percent points over the baseline. Data sets are ordered by ascending hubness ($S^{k=10}$) as measured before any hubness reduction. **a** Successful methods. **b** Methods with mixed results

### Details of hubness reduction and classification performance

Figure 4 depicts the evaluation results in greater detail for six methods representing the different families of hubness reduction methods described in Sect. 3. Data sets in this plots are again ordered by ascending hubness ($S^{k=10}$) as measured before any hubness reduction, with low hubness data sets in the upper rows and high hubness data sets in the lower rows. Measures are absolute differences after and before hubness reduction.

We selected three methods achieving low ranks in both hubness reduction and classification performance (Fig. 4a). LS, MP, and DSL represent local scaling, global scaling, and density gradient flattening, respectively. Looking at the hubness reduction results, depicted as absolute changes in hubness in the left part of Fig. 4a, we can see that all three methods show only very small improvements for low hubness hubness data sets, but do show substantial hubness reduction for higher hubness data sets, starting around data set 26 (lungcancer), which exhibited hubness of 1.2483 before reduction. Previous studies [49] on hubness reduction have shown a similar picture with reduction methods being effective above 10-occurrence skewness of 1.4. All three methods show highly comparable hubness reduction in general, with MP (dark gray bars) achieving negligibly stronger hubness reduction than LS (light gray bars) in many data sets, explaining the nonsignificant rank differences in Fig. 3a. DisSim$^\mathrm{Local}$ (DSL, black bars) performs equally well as LS and MP on many data sets. Hubness reduction is nearly always on par with both scaling methods. Though DSL actually increases hubness for data sets 35 and 39 (farm ads, dorothea), coinciding with degraded accuracy, it achieves lowest hubness values for many others, resulting in the best rank in terms of hubness reduction in Fig. 3a.

Looking at absolute changes in classification performance in the right part of Fig. 4a, most significant changes are observed in high hubness data sets, starting around data set 25 (splice). Exceptions are two data sets of low to medium hubness (chronic kidney disease, no. 7, duke, no. 18), where DSL considerably degrades classification performance. On the other hand, LS increases classification performance for many of the high hubness data sets, with MP showing highly similar results. While LS often shows slightly improved classification accuracy compared to MP, these differences are nevertheless nonsignificant as already shown in Fig. 3b. DSL shows competitive classification performance compared to scaling methods for some high hubness data sets but is more often detrimental (e.g., farm ads, no. 35, dorothea, no. 39, CNAE-9, no. 40, sector, no. 47). While the degradation in classification for DSL coincides with an increase in hubness for data sets 35 and 39, there is no such connection for all other cases.

Overall, both scaling methods MP and LS consistently reduce hubness and often improve classification performance. They may safely be applied on data sets from low to high hubness, without any noticeable risk of performance degradation. DSL is sometimes competitive with LS and MP, but also shows a higher risk of decreased performance measures, hinting at density gradient flattening being less generally applicable than scaling.

Figure 4b shows three methods with mixed evaluation results. LCENT, $\ell ^p$ norms, and SNN represent centering techniques, choosing alternative distance measures, and shared neighbors approaches. Looking at the hubness reduction results, depicted as absolute changes in hubness in the left part of Fig. 4b, LCENT (light gray bars) shows increased hubness for almost all data sets, except the highest hubness data sets 49 (c1ka-twitter) and 50 (dna). Shared nearest neighbors (SNN, black bars) on the other hand is able to reduce hubness in all medium to high hubness data sets. Application of $\ell ^p$ norms (LP, dark gray bars) has mixed effects on high hubness data, with reduction of hubness for data sets 42 (DMOZ), 43 (Amazon commerce reviews) and 49 (c1ka-twitter), and considerable increase in hubness for data sets 35 (farm ads), 36 (gisette), 39 (dorothea) and 40 (CNAE-9).

Looking at absolute changes in classification performance in the right part of Fig. 4b, it can be seen that almost only LCENT (light gray bars) is able to improve accuracy on selected data sets, typically for those from the text domain, for example data sets 43 (Amazon commerce reviews) and 49 (twitter). Looking at the respective hubness performance, hubness is reduced in data set 49, but, strikingly, it is actually strongly increased in data set 43. LCENT can be a suitable choice for data sets from the text domain, with scaling methods LS and MP (see right side in Fig. 4a) still performing better. Choosing alternative $\ell ^p$ norms (LP, dark gray bars) or shared nearest neighbors (SNN, black bars) shows virtually only degraded classification performance, especially for high hubness data. Hubness reduction with these methods does not seem to be sensible.

**Fig. 5:**
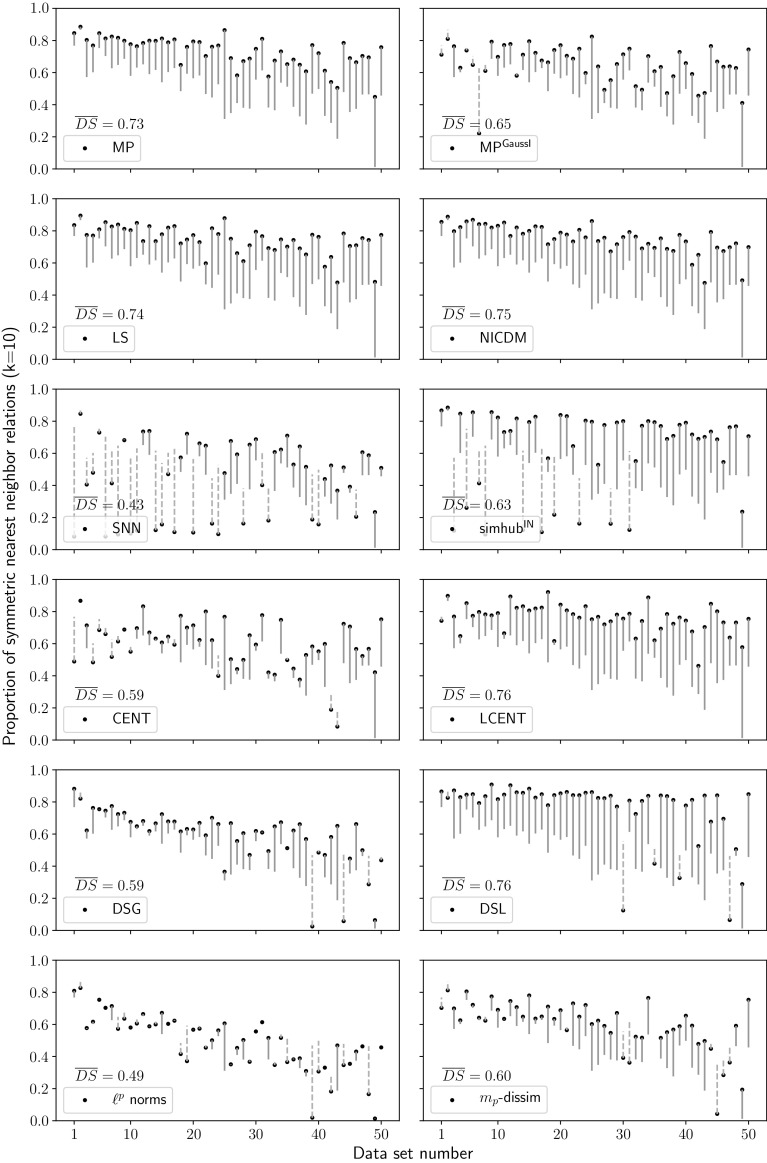
Symmetric neighborhoods. Percentage of symmetric nearest neighbor relations ($k=10$). Solid lines (violet) indicate improvement over the baseline, dashed lines (orange) indicate degradation. Filled circles represent symmetry using the indicated method, while the opposite end of the line represents the baseline which is identical for all subplots. $\overline{DS}$ denotes the average degree of symmetry. Baseline $\overline{DS} = 0.50$ (color figure online)

### Neighborhood symmetry

Hubness negatively affects neighborhood symmetry as outlined in Sect. 2.1. Hubness reduction should therefore increase the proportion of symmetric *k*-nearest neighbor relations. Figure 5 depicts changes in neighborhood symmetry after hubness reduction compared to baseline in twelve subplots for the twelve hubness reduction methods. To explain what is shown in these twelve subplots, we first turn to the results for mutual proximity (MP), in the left subplot of the top row in Fig. 5. We show the percentage of symmetric nearest neighbor relations ($k=10$) on the y-axis for all 50 data sets on the x-axis. To be more precise, we show the change in percentage of symmetric nearest neighbor relations when MP is applied relative to a baseline when no hubness reduction is done. These changes are shown as solid violet lines in case the percentage of symmetric nearest neighbor relations increases due to hubness reduction, and as a dashed orange line in case it decreases. Please note that the dot at one end of every line signifies the result achieved by MP, whereas the other end of a line signifies the baseline result. These ends show a clear trend of decreasing values from left to right: Since the data sets on the x-axis are ordered by their hubness values (refer to Table 3), this indicates that the baseline measures create fewer symmetric nearest neighbor relations in data sets with higher hubness, as expected from theory. These baseline values are identical in all subplots. The subplot for MP clearly shows that the percentage of symmetric nearest neighbor relations is improved for all data sets. This improvement is stronger for high hubness data sets. On average, the proportion of symmetric relations $\overline{DS}$ increased from baseline 0.50 to 0.73 when using MP.

As a matter of fact, all local and global scaling methods consistently increase the number of symmetric neighborhood relations in nearly all cases (MP: $\overline{DS}$=0.73, MP$^\mathrm{GaussI}$: $\overline{DS}$=0.65, LS: $\overline{DS}$=0.74, NICDM: $\overline{DS}$=0.75). They show comparable results among each other with the effect being weaker in case of MP$^\mathrm{GaussI}$ for some data sets. Modeling distance distributions with independent Gaussians may not approximate the real distributions in these cases. LCENT and DSL also improve neighborhood symmetry to a similar extent as scaling methods (both $\overline{DS}$=0.76). DSL achieves the highest symmetries for several data sets, but reduces symmetry in five cases, for example, data sets 35 (farm ads) and 39 (dorothea), which in addition also show increased hubness after application of DSL (cf. Fig. 4a). The global variants of centering (CENT) and DisSim (DSG) are on average much less successful in increasing symmetry (both $\overline{DS}=0.59$). This is not surprising, as they were argued to reduce hubness in unimodal distributions, but real-world data sets are usually better described as mixtures of distributions. It is noteworthy that most cases of improved symmetry due to centering correspond to data sets from the text domain. Usage of alternative $\ell ^p$ norms changes symmetry compared to baseline only for a few data sets (LP: $\overline{DS}$=0.49). Indeed, in some cases parameter selection yielded $p=2$, reducing the metric to the Euclidean baseline. Small positive effects were found for the $m_p$-dissimilarity ($\overline{DS}$=0.60). SNN severely reduces symmetry for many data sets, predominantly among those with low to medium hubness, resulting in a reduced $\overline{DS}$ of 0.43. The SNN-variant simhub$^\mathrm{IN}$ also reduces neighborhood symmetry in several cases. However, it does increase symmetry in data sets with high hubness strongly, and on average to 0.63.

The results in this section are based on neighborhood sizes $k=10$. Similar effects on neighborhood symmetry were observed with neighborhood ranges adaptive to data set sizes using $k=\frac{n}{10}$ (not shown).

**Fig. 6:**
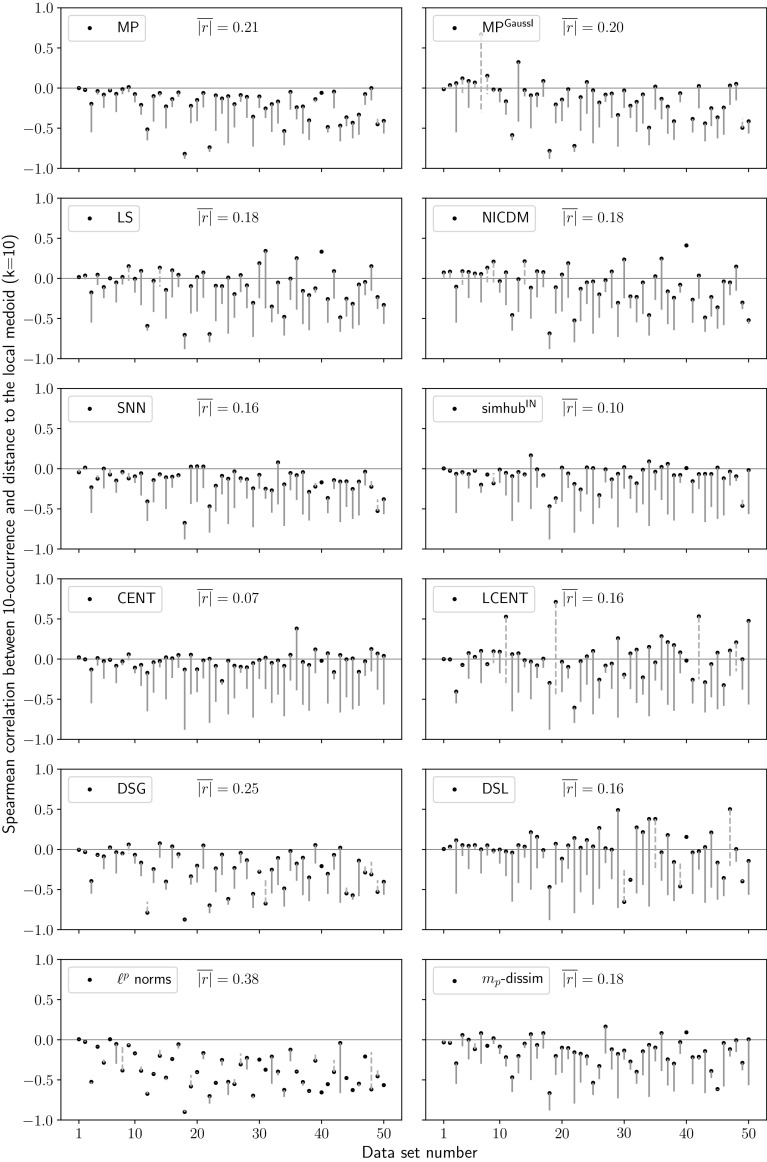
Local medoids. Spearman correlation between the 10-occurrence of each object and the distance to their local medoids (k=10). Improvements and degradations are indicated analogously to Fig. 5. Changes are considered to be improvements, if the absolute value of correlation is reduced. $\overline{|r|}$ denotes the average of absolute values of correlation after hubness reduction. Baseline $\overline{|r|}=0.38$

### Similarity to centroids

Objects highly similar to their (local) centroids may emerge as hubs (cf. Sect. 2.1). Reducing these similarities should therefore improve hubness. Centroids can be trivially computed from vector data or may be derived from distances based on metrics like the Euclidean norm. It is, however, not generally possible to calculate them from arbitrary dissimilarity matrices. We therefore use (local) medoids as proxies for their corresponding centroids, and measure correlation between *k*-occurrence and distance to the medoid. Reduced correlation should hint at reduced emergence of hubs.

Figure 6 depicts changes in correlation after hubness reduction compared to baseline analogously to Fig. 5 displaying neighborhood symmetry changes. To describe the plot in more detail, let us consider the left subplot in the fourth row, showing the results for CENT. Dot markers indicate the correlation between 10-occurrence and distance to local medoids on the y-axis for each data set on the x-axis. As expected, there is a trend of stronger negative correlations in data sets of higher hubness before hubness reduction. The average absolute value of correlation is denoted as $\overline{|r|}$ and given for all hubness reduction methods in their corresponding subplot. The horizontal line indicates the targeted correlation value of $r=0$. CENT uses the inner product as similarity measure. After centering, the data centroid is a zero vector and, thus, all inner product similarities to the centroid are uniform zero [56]. Consequently, there is no correlation between these similarities and *k*-occurrence. Given the results of CENT with most correlations being very close to zero and the average correlation $\overline{|r|}=0.07$, we assume that medoids are indeed suitable proxies for centroids.

Vertical lines reveal the change in correlation due to hubness reduction compared to baseline. That is, baseline correlations reside at the end of the lines opposite to the dot markers. Solid violet lines indicate improved correlations, which are closer to zero after hubness reduction. Dashed orange lines signalize degraded correlations with higher absolute values after hubness reduction. Consider for example the right subplot of the fourth row: In five cases, LCENT ‘overshoots‘ and yields positive correlations of higher absolute value than the negative correlations before hubness reduction.

We find an average Spearman $\overline{|r|}=0.38$ for the baseline not using any hubness reduction. All reduction methods are able to reduce this correlation. Weakest average correlations ($\overline{|r|}<0.2$) are observed for LCENT, DSL, LS, NICDM and simhub$^\mathrm{IN}$. All other methods, except for $\ell ^p$ norms ($\overline{|r|}=0.38$), create weak correlations as well. In case of SNN, this may be partly due to the fact, that its secondary distances can only take a low number of different discrete values (precisely the neighborhood size $k+1$). Consequently, SNN often yields many equal distances, and random rankings among those distances reduce correlation.

Interestingly, the correlations are reduced not only when using spatial centrality-based methods, but also for those based on neighborhood symmetry, like for example MP or LS.

**Fig. 7:**
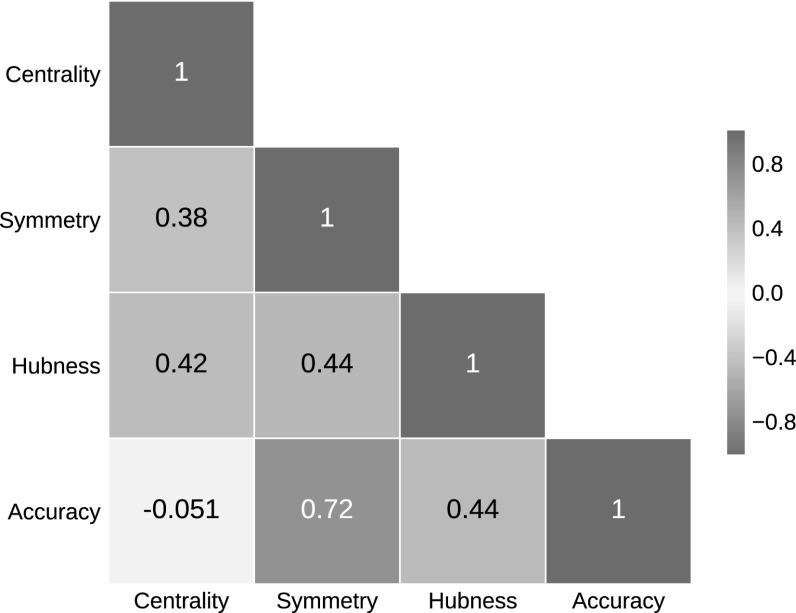
Spearman correlation between evaluation criteria. Correlations are calculated from the ranks of twelve hubness reduction methods and two baseline measures given each criterion (see Sect. 5.5). ‘Centrality’ refers to the association between *k*-occurrence and distance to local medoids (Sect. 5.4), ‘Symmetry’ refers to the proportion of symmetric relations in nearest neighbor lists (Sect. 5.3), ‘Hubness’ is the skewness of the 10-occurrence distribution, and ‘Accuracy’ denotes the classification performance

### Association of evaluation criteria

We rank all methods and baseline measures by their success in hubness reduction (Fig. 3a), classification performance (Fig. 3b), degree of symmetric neighborhood relations (Fig. 5), and spatial centrality reduction (Fig. 6). Across all hubness reduction methods and baseline measures, symmetry ranks are highly correlated with accuracy ranks (Spearman $r=0.72$, Fig. 7). That is, effectiveness in terms of increased symmetry corresponds well to the results in nearest neighbor classification. Ranks in reducing spatial centrality are more in line with the ranks in hubness reduction (Spearman $r=0.42$, Fig. 7), than with classification accuracy, for which we observe no rank correlation with spatial centrality ($r=-0.05$). The association between reduced spatial centrality and hubness reduction is, however, less clear than the association between strengthened neighborhood symmetry and increased classification performance. To give one concrete example, correlation between 10-occurrence and distance to local medoids is strongly decreased using CENT, but hubness is reduced only slightly, and in case of MP, vice versa (Fig. 6).

**Fig. 8:**
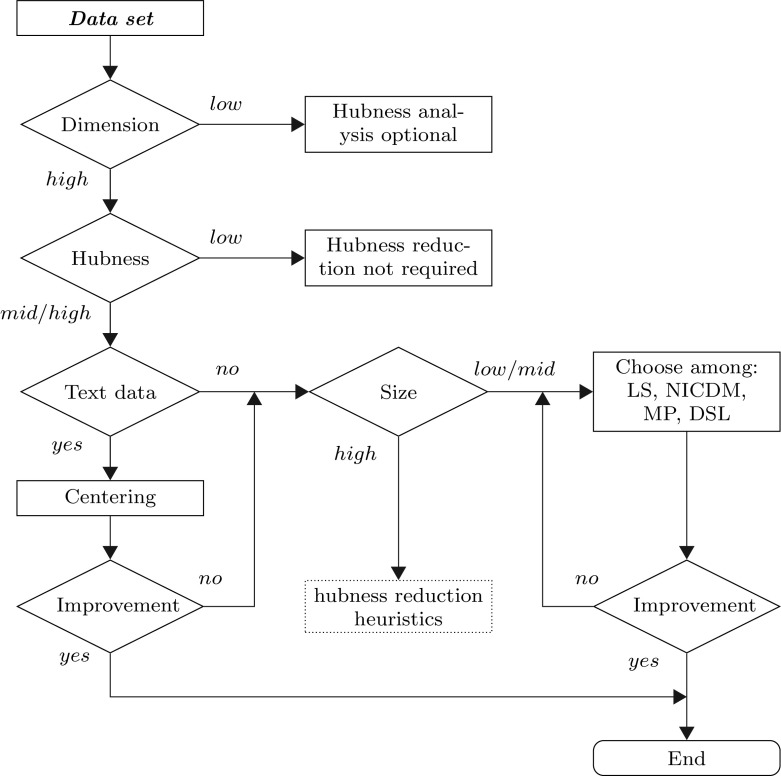
Choosing appropriate hubness reduction methods

## Discussion

We find that global and local scaling methods (MP, LS, NICDM) consistently improve performance for all evaluation measures over a wide range of data sets from various domains. This result is in line with the findings of a previous study [49]. Scaling methods achieve highest classification accuracy among the competing hubness reduction methods, and perform competitively to the best methods given the other evaluation measures. LS and NICDM are conceptually similar. While LS only considers the distance to one fixed neighbor, NICDM uses statistics over several neighbors. For this reason, we had expected higher stability of NICDM results over LS. Instead, both methods perform equally well. Carefully tuning their hyperparameters might have compensated for any instability. Density gradient flattening with DSL yields the best results in hubness reduction and neighborhood symmetry. Differences in classification performance between DSL and scaling methods are nonsignificant. We recommend using any of the four methods MP, LS, NICDM, and DSL for general hubness reduction. For large data sets, the cubic time complexity of MP is prohibitive, whereas LS, NICDM, and DSL scale only quadratically with the data set size. In the framework of mutual proximity, quadratic complexity can be achieved by approximating the empiric distance distribution with normal or Gamma distributions [49]. The approximation with normal distributions (MP$^\mathrm{GaussI}$) yielded good results. Overall performance degradation compared to MP using empiric distributions is not significant, and might be caused by some data sets, for which independent Gaussians do not fit the true distance distributions well. MP$^\mathrm{GaussI}$ may thus be used, when MP is too expensive in large data sets. Furthermore, distance distributions can be modeled using any continuous distribution. Given specific domain knowledge, other distributions may yield better MP approximations.

For very large data sets, algorithms with quadratic complexities are not applicable. Hubness reduction heuristics with subquadratic time and space requirements can be devised: LS, NICDM, and DSL require local neighborhood information. Their transformations could be accelerated using approximate nearest neighbor techniques. *Locality-sensitive hashing* [31] is commonly used for approximate search in high-dimensional spaces [1]. Since LSH requires vector data, different approaches are necessary for data sets only providing distances between objects. Sampling strategies could serve as an alternative for these cases, and may also be employed to reduce the complexity of mutual proximity. Heuristics based on these or other strategies would allow for hubness reduction in very large data sets. Evaluation of effectiveness and efficiency is yet to be performed, however.

Centering approaches show mixed results. They improve performance measures in several data sets from the text domain. Several other data sets are hardly influenced by centering, possibly due to its global nature: Hubs emerge close to the global mean only in case of unimodal data distributions [44]. Subtracting the mean does not eliminate spatial centrality in data sets with underlying multimodal distributions. The mechanism of hubness reduction behind centering does fail in such cases. LCENT similarities are calculated by subtracting *affinity* to local centroids. However, significant improvements over CENT were not observed in the evaluation. Centering seems to be applicable primarily to text data sets, but it does not outperform scaling methods or DSL in these cases. Due to its low cost, CENT can be applied to large data sets, and may thus be used for hubness reduction, when other methods are too expensive.

We do not recommend the other evaluated methods for hubness reduction. Using alternative $\ell ^p$ norms or DSG does not yield improved performance measures. The formulation of DSG assumes all data to be generated from a unimodal probability distribution, an assumption presumably violated by many real-world data sets used in this study. This could explain the marked performance difference between the global and local DisSim variants, since DSL can handle mixtures of distributions by considering local neighborhoods. The shared neighbors approaches SNN and simhub$^\mathrm{IN}$ reduce hubness, but fail to preserve, let alone improve data semantics. It has been argued previously that shared neighbor transformations cause information loss, because they only use rank information, and their codomain contains only $k+1$ different values [20]. The data-dependent $m_p$-dissimilarity measure improves performance indicators compared to baseline, but does not yield results competitive with the best methods.

Given the results in Figs. 5, 6, 7, we observe that symmetric nearest neighbor relations have a stronger influence on classification performance than distances to medoids, which weakly corresponds to hubness. A possible explanation for these observations is that spatial centrality might not be the actual (or at least not the only) driving force in hub emergence. For example, Low et al. describe hubness as an effect of density gradients due to non-uniform data distributions or boundary effects [39]. Spatial centrality-based methods may fail to reduce hubness and improve data semantics in such cases, unless they also flatten the density gradient. As opposed to this, asymmetric nearest neighbor relations are not a *source* of hubness, but a necessary *consequence* of skewed *k*-occurrences. Fixing detrimental effects of hubness one step later in the chain of causation might give methods based on neighborhood symmetry the advantage of independence of the primary cause of hubness. Verifying this hypothesis remains a task for future research.

Figure 8 serves as a reference for the interested reader. Based on some simple criteria, it guides through the hubness analysis workflow: Dimensionality and hubness measurements help decide, whether hubness reduction is indicated for given data. Data set size and application domain help decide which method to choose. Finally, alternatives are recommended if the selected hubness reduction method does not yield sufficient performance improvements.

On a side note, the difference between LCENT and LS has previously been described in terms of their theoretical motivation [27]: LCENT tries to reduce correlation between *k*-occurrence and local affinity, which is related to the distance to the local centroid/medoid. On the other hand, LS tries to make nearest neighbor relations more symmetric. Our empirical results do not support this distinction, since both methods achieve similar improvements both in terms of neighborhood symmetry and *k*-occurrence/medoid correlation.

## Conclusion

In this paper, we presented a large-scale empirical evaluation of unsupervised hubness reduction methods. We analyzed hubness in terms of *k*-occurrence skewness, spatial centrality, and neighborhood symmetry before and after hubness reduction. Global and local scaling (MP, LS, NICDM) as well as density gradient flattening (DSL) improve these measures as well as data semantics (classification accuracy) over a wide range of data sets from various domains and may be considered state-of-the-art in hubness reduction. Global and localized centering are not as generally applicable, but can be successful for data from the text domain, with CENT being especially simple and inexpensive.

Future work will continue to investigate the impact of hubness on supervised and unsupervised learning methods beyond nearest neighbor classification. The development and evaluation of hubness reduction heuristics with subquadratic time and space complexity will allow to tackle large data sets.

## Footnotes

1 Note that centering requires only $\mathcal {O}(n m)$ time, if no subsequent distance calculations are performed.

2 https://pypi.python.org/pypi/hub-toolbox/.

3 https://github.com/OFAI/hub-toolbox-python3.

4 https://github.com/OFAI/hub-toolbox-matlab.
